# Improving CRISPR‐Cas‐mediated RNA targeting and gene editing using SPLCV replicon‐based expression vectors in *Nicotiana benthamiana*

**DOI:** 10.1111/pbi.13384

**Published:** 2020-04-28

**Authors:** Yicheng Yu, Xiao Wang, Houjun Sun, Qiang Liang, Weichi Wang, Chengling Zhang, Xiaofeng Bian, Qinghe Cao, Qiang Li, Yiping Xie, Daifu Ma, Zongyun Li, Jian Sun

**Affiliations:** ^1^ Jiangsu Key Laboratory of Phylogenomics and Comparative Genomics School of Life Sciences Jiangsu Normal University Xuzhou Jiangsu China; ^2^ Xuzhou Institute of Agricultural Sciences in Jiangsu Xuhuai District Xuzhou Jiangsu China; ^3^ Institute of Food Crops Provincial Key Laboratory of Agrobiology Jiangsu Academy of Agricultural Sciences Nanjing Jiangsu China

**Keywords:** LwaCas13a, SpCas9, LbCas12a, deconstructed SPLCV strategy, *Nicotiana benthamiana*, RNA targeting and gene editing efficiency

Geminiviruses are a family of plant viruses with circular single‐stranded DNA genomes. They have been deconstructed by researchers for multiple biotechnological applications, including protein expression, gene silencing and genome editing, in plants (Lozano‐Durán, 2016). Under the deconstructed virus strategy, the coat protein and movement protein genes were removed from the geminiviruses, while the sequences required for replication were retained. The replicons replicate after delivery to plant cells and increase the copy number of carried DNA; this leads to high levels of gene expression (Lozano‐Durán, 2016). Although a few geminiviral replicon‐based vectors have been used in gene targeting (Baltes *et al.*, 2014; Cermak *et al.*, 2015; Wang *et al.*, 2017), the list of DNA replicon‐based vectors is still limited in plants, especially for food crops. In this study, we developed *sweet potato leaf curl virus* (SPLCV) replicon‐based expression vectors. We tested the efficiency of these vectors in CRISPR‐Cas‐mediated RNA targeting and gene editing by using *Nicotiana benthamiana* as model plant.

SPLCV is a monopartite geminivirus belonging to the genus *Begomoviruses* (Bi and Zhang, 2012). The coding region of SPLCV‐JS (accession number: KF040468.1) replication‐associated proteins (Rep:1900 bp, encoding four proteins: AC1, AC2, AC3 and AC4) and intergenic region (IR:284 bp) were synthesized and cloned into the binary vector pCambia0390 together with GFP expression cassette (U4:GFP) in an IR‐GFP‐Rep‐IR origination to produce the reporter vector SPLCV‐GFP (Figure 1a). A regular T‐DNA vector (T‐GFP) harbouring the same expression cassette was used as control. *N. benthamiana* leaves infiltrated with *Agrobacterium tumefaciens* containing SPLCV‐GFP significantly showed stronger GFP fluorescence than T‐GFP (Figure 1b). We confirmed the circularization between the two IRs by PCR with a prime pair facing opposite directions in the SPLCV‐GFP‐infiltrated leaves (Figure 1c). The GFP transcripts in SPLCV‐GFP‐infiltrated leaves were 22.5 times higher than those in T‐GFP (Figure 1d).

**Figure 1:**
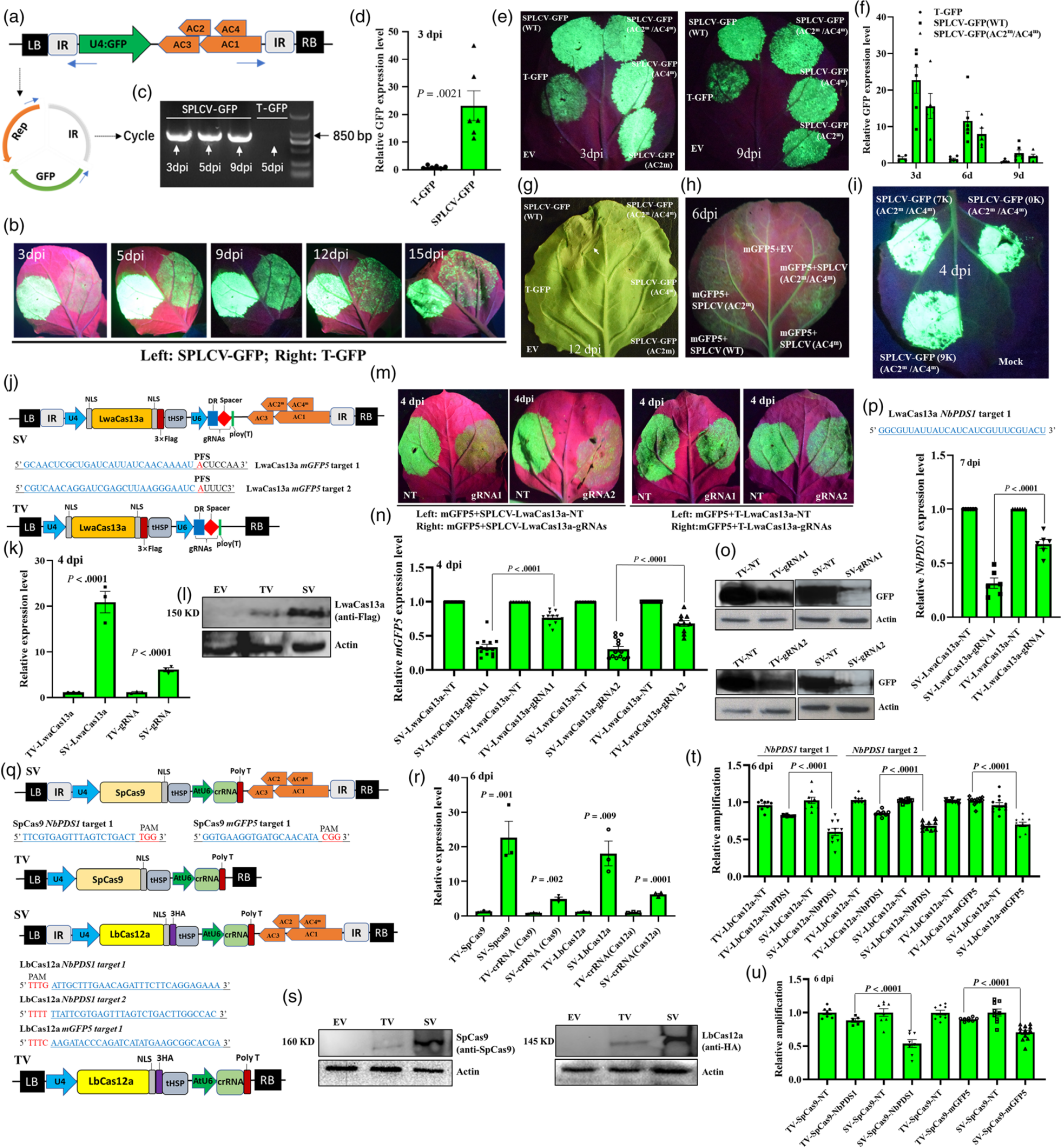
Improving CRISPR‐Cas‐mediated RNA targeting and gene editing using SPLCV replicon‐based expression vectors in *Nicotiana benthamiana*. (a) Schematic of the SPLCV‐GFP construct. DNA replicon is presented as a circle. (b) Time course of GFP expression in *N. benthamiana* leaves infiltrated with SPLCV‐GFP (left side) or regular vector (right side; T‐GFP). (c) Confirmation of the SPLCV replicon in *N. benthamiana* leaves. Genomic DNA was amplified by PCR using outward‐facing primers. (d) Quantification of GFP transcripts. (e) GFP fluorescence in *N. benthamiana* leaves infiltrated with wild type (WT) and mutational SPLCV‐GFP vectors, namely AC4^m^, AC2^m^ and AC2^m^/AC4^m^. (f) Quantification of GFP transcripts in WT‐ and AC2^m^/AC4^m^‐infiltrated leaves. (g) Phenotype of cell necrosis after 12 days of infiltration of various GFP vectors. (h) Representative images (out of three) showing RSS activity in various viral vectors as indicated by GFP silencing experiments. (i) Effect of size on AC2^m^/AC4^m^‐mediated GFP expression in *N. benthamiana* leaves. (j) Schematic of the LwaCas13a vectors used to evaluate *mGFP5* targeting activity (SPLCV‐based vector: SV; regular vector: TV). (k) Expression levels of LwaCas13a and gRNAs (target 1) in *N. benthamiana* leaves infiltrated with SV and TV. (l) Western blot detection of the LwaCas13a protein. (m) Representative images showing *mGFP5* knockdown by using SVs and TVs. The left and right sides of leaves were infiltrated with mGFP5 and non‐targeting (NT) vectors or mGFP5 and targeting vectors, respectively. (n) Quantification of *mGFP5* expression level. (o) Representative Western blot results showing mGFP5 accumulation. (p) Targeting of an endogenous transcript *NbPDS1* using SV and TV. (q) Schematic of the SpCas9/LbCas12a vectors used to evaluate gene editing efficiency. (r) Expression levels of SpCas9/LbCas12a and crRNAs (*NbPDS1* target 1) in SV‐ and TV‐infiltrated *N. benthamiana* leaves. (s) Western blot detection of the SpCas9/LbCas12a protein. (t‐u) Detection of the mutant frequencies in different targets by qPCR. The relative amplification was calculated by setting the expression of NT‐infiltrated samples as 1.0.

After several days of infiltration, evident necrosis was found in most SPLCV‐GFP‐infiltrated leaves. To decrease cell lethality, three mutational SPLCV vectors were constructed: (i) AC4^m^: a T‐to‐A mutation was introduced in the coding region of Rep, thereby resulting in a premature stop of translation of AC4 (AC4:26T→A(Leu9TAA); AC1:183T→A(Leu61Leu)); (ii) AC2^m^: a premature termination codon mutation was introduced in the coding region of AC2, and this mutation changed one amino acid of AC1 (AC2:31A→T(Lys11TAG); AC1:1034A→T(Glu345Val)); and (iii) AC2^m^/AC4^m^ double mutant. All constructs were sequenced to confirm the correct mutation sites. The GFP expression cassette was cloned into the three mutational vectors, and the expression efficiency was compared with that of SPLCV‐GFP (WT). No visible decrease in GFP fluorescence was observed in *N. benthamiana* leaves infiltrated with *A. tumefaciens* containing these mutational constructs at days 3 and 9 (Figure 1e). The GFP transcripts exhibited no significant difference between the WT and AC2^m^/AC4^m^ vectors (Figure 1f). These mutational constructs did not induce necrosis at day 12 (Figure 1g). We then tested the RNA‐silencing suppressor (RSS) activity of these basic viral vectors through a GFP silencing experiment in *N. benthamiana* (16C) leaves. Figure 1h shows that GFP silencing was only observed in empty and AC2^m^/AC4^m^ vectors. This result implied that the AC2^m^/AC4^m^ construct lost RSS activity. Insertion of up to 9 kb noncoding DNA sequence in AC2^m^/AC4^m^ still resulted in strong GFP expression (Figure 1i). This result suggested that the cargo capacity of SPLCV was sufficient to deliver CRISPR nucleases.

We then tested whether the SPLCV vector can enhance LwaCas13a‐mediated RNA targeting activity in plants (Abudayyeh *et al.*, 2017). The AC2^m^/AC4^m^ construct was used for this series of experiments. We artificially synthesized the DNA sequence of plant codon‐optimized LwaCas13a and cloned into AC2^m^/AC4^m^ and regular vectors with 3×Flag fusion on the C terminus and a dual‐flanking nuclear localization sequence (NLS) under the expression of the U4 promotor. Moreover, we designed two gRNAs against the *mGFP5* transcript and recombined the targeting vectors (SPLCV‐based vector: SV; T‐DNA vector: TV; Figure 1j). The same vectors containing a non‐targeting (NT) spacer were used as control. After 4 days of infiltration, a higher accumulation of LwaCas13a and gRNA transcripts and the LwaCas13a protein was found in SV‐infiltrated leaves than in TV‐infiltrated ones (Figure 1k and l). In comparison with the left side of leaves infiltrated with mGFP5/NT constructs, a visible decline in GFP fluorescence was observed in the corresponding right side infiltrated with SVs at day 4. This phenomenon was not apparent in TVs (Figure 1m). The SVs resulted in significantly higher levels of knockdown of *mGFP5* (67% for gRNA1, 70% for gRNA2) than TVs (25% for gRNA1, 32% for gRNA2; Figure 1n). The SVs‐infiltrated samples accumulated less mGFP5 protein than TVs (Figure 1o). In addition, we obtained the similar results when targeting an endogenous transcript *NbPDS1* (Figure 1p). These results clearly showed that SPLCV‐based RNA‐targeting vectors were more efficient than regular ones.

We further tested whether SPLCV vector can enhance SpCas9‐ and LbCas12a‐mediated gene editing in plants. The AC4^m^‐derived constructs could be efficient for gene editing in plants due to the loss of cell lethality but maintained RSS activity (Figure 1h; Mao *et al.*, 2018). Thus, the AC4^m^ vector was used in this series of experiments. We cloned plant codon‐optimized SpCas9 (with NLS fusion on the C terminus) and LbCas12a (with 3×HA and NLS fusion on the C terminus) into AC4^m^ and regular vectors under the expression of the U4 promotor. We designed several crRNAs against *NbPDS1* and *mGFP5* in wild type and 16C *N. benthamiana*, respectively, and recombined the SVs and TVs (Figure 1q). The same vectors containing NT crRNA were used as control. After 6 days of infiltration, a higher accumulation of nucleases, crRNA transcripts and the nuclease protein was found in SV‐infiltrated leaves than in TV‐infiltrated ones (Figures 1r and s). The average relative amplification values of the target sites obtained using the qPCR‐based method were approximately 1.01 and 0.90 in NT‐infiltrated and TV‐infiltrated samples, respectively (Figures 1t and u; Peng *et al.*, 2018). Such a notion suggests that the mutation frequency for TVs was approximately 10%, and this result was similar to a previous report (Bernabé‐Orts *et al.*, 2019). However, the average relative amplification values of five target sites were approximately 0.65 in SV‐infiltrated samples (Figures 1t and u). Thus, the mutation frequencies for SVs were 35% and much higher than those of TVs.

In summary, our work expands the existing list of geminiviral replicon‐based vectors in plants. The SPLCV‐based vectors offer an opportunity to enhance the efficiency of CRISPR‐Cas‐mediated gene knockdown, knockout and knockin in plants, especially for its host sweet potato; gene editing through de novo induction of meristems may also be conducted in this important hexaploid crop in the near future (Maher *et al.*, 2020).

## Conflict of interests

The authors have no conflict of interests.

## Author contributions

J.S. and Z.L. designed experiments; Y.Y., X.W., H.S., Q.L., W.W., C.Z. and X.B. performed experiments; Q.L., Q.C., Y.X. and D.M. provided suggestions. J.S. wrote the paper.
